# Reference values and biological factors influencing skin autofluorescence

**DOI:** 10.3389/fendo.2025.1700892

**Published:** 2025-11-06

**Authors:** Bruce H.R. Wolffenbuttel, Henderikus E. Boersma, Robert van Waateringe, Andrew D. Paterson, Melanie M. van der Klauw

**Affiliations:** 1 Department of Endocrinology, University of Groningen, University Medical Center Groningen, Groningen, Netherlands; 2 Department of Internal Medicine, University of Groningen, University Medical Center Groningen, Groningen, Netherlands; 3 Program in Genetics and Genome Biology, Hospital for Sick Children, Toronto, ON, Canada; 4 Divisions of Biostatistics and Epidemiology, Dalla Lana School of Public Health, University of Toronto, Toronto, ON, Canada

**Keywords:** advanced glycation end-products, skin autofluorescence, reference values, diabetes mellitus, smoking, physical activity, cardiovascular disease, renal dysfunction

## Abstract

**Introduction:**

Tissue glycation, measured as skin autofluorescence (SAF) with an AGE Reader, has been associated with incident type 2 diabetes (T2D), cardiovascular disease (CVD), and CVD and cancer mortality. Validated reference intervals in healthy individuals are needed to make useful and precise risk estimations.

**Methodology:**

This study utilises data from 82,870 participants of Western European descent in the Lifelines Cohort Study, a population-based study in the Netherlands. Reference values for SAF were established in healthy participants. Decade-specific mean SAF values were evaluated for former and current smokers, T2D, CVD, and impaired renal function. We also assessed the association of regular physical activity (PA) with SAF scores.

**Results:**

Reference values for healthy individuals were established in 8,179 men and 7,930 women between 18 and 70 years, who had never smoked, had a BMI below 35 kg/m^2^, and did not meet the metabolic syndrome criteria. Linear regression analyses yielded the following prediction for SAF, separately by sex: in males, SAF predicted = (0.0191 × age) + 1.038, and in females, SAF predicted = (0.0188 × age) + 0.994. Current smokers had consistently higher SAF scores. There was a progressively higher SAF with a higher number of pack-years of smoking in each age decade, in both sexes. For each decade, both people with T2D and CVD had significantly higher SAF values compared to healthy individuals. The same applied to participants with impaired renal function. There was a complex non-linear relationship between PA and age-adjusted SAF score: mean SAF was the highest in sedentary participants (e.g., 0 min/week moderate-to-vigorous physical activity), lowest in those with 150–299 min/week PA, and gradually increasing in participants with higher than 600 min/week PA.

**Conclusions:**

We provide robust reference values for SAF, established in healthy individuals of Western European descent, separately by sex, who have never smoked. Higher levels of SAF are observed in former and current smokers and in people with T2D, CVD, and impaired renal function. The relationship between physical activity and SAF scores is complex, with higher SAF scores demonstrated in sedentary people and those performing a large amount of moderate-to-vigorous physical exercise.

## 1 Introduction

Advanced glycation end-products (AGEs) are a heterogeneous group of compounds, which are formed through the process of non-enzymatic glycation of proteins, lipids, and nucleotides (1–5). Glycation has several consequences for molecules that interact with AGEs, leading to increased tissue and vascular stiffness, making lipoprotein particles more atherogenic, and reducing contractility and function of the heart. It has been shown that AGEs accumulate with increasing age. Furthermore, endogenous accumulation increases with increasing blood glucose concentrations, higher oxidative stress levels, and chronic inflammation (3, 4). In addition, exogenous AGEs are ingested from our diet or inhaled through cigarette smoking, resulting in AGE accumulation (6, 7). AGEs that accumulate in tissues are cleared very slowly. Decreased renal function reduces the clearance of AGEs from the body, increasing their accumulation (8).

Clinically, accumulation of AGEs is associated with a higher rate of developing diabetes-related complications and atherosclerosis (9, 10), and they are central in the increased cardiovascular morbidity and mortality which can be observed in people with either type 1 or type 2 diabetes (11, 12).

We can relatively easily measure the cutaneous accumulation of AGEs with non-invasive techniques (13–15). A device called the “AGE Reader” uses the capability of several accumulated AGEs to emit fluorescence, and can assess the degree of AGE accumulation in the skin by measuring skin autofluorescence (SAF) at specific wavelengths after exposure to light. Thus, SAF provides an integrated measure of the accumulation of AGEs. A wealth of studies has shown that several factors influence SAF in addition to age, such as smoking habits, average glycaemia, renal function, and caffeine consumption (8, 16–18). SAF is associated with the presence of the so-called metabolic syndrome—a well-known cluster of cardiometabolic risk factors—and some of its individual components (18, 19). Similarly, an association was reported between both white blood cell count and SAF (20), as well as between the presence of anaemia and SAF, possibly in relation to the presence of low-grade inflammation (21). Cessation of smoking results in a gradual decrease of SAF, which slowly drops to age-conforming values after only 12 to 15 years (22). After the initial report that a single-nucleotide polymorphism in NAT2 strongly influences SAF measurements (23), several other genetic loci influencing SAF have been reported (24).

Several studies have shown that SAF is strongly associated with future disease events. Using data obtained in the Lifelines Cohort Study, we observed a significant association between higher SAF and an increased risk of type 2 diabetes, cardiovascular events, and all-cause mortality (25). In a follow-up study, combining data from Lifelines with data obtained from death certificates, we have reported that higher SAF was associated with specific cardiovascular and cancer mortality (26). This relationship was independent of other risk factors. Multiple smaller studies targeting specific disease populations (i.e., in people with type 1 or type 2 diabetes or end-stage kidney disease) found significant associations between SAF and heart disease, peripheral vascular disease, cardiovascular mortality, and all-cause mortality (27–32). Furthermore, higher SAF is associated with a higher prevalence of traditional cardiovascular risk factors, such as elevated LDL-cholesterol and elevated blood pressure (33).

Several of these studies have used reference data for SAF supplied by the manufacturer, and these reference data were based on measurements obtained from a small group of 428 healthy individuals (15, 34). Many participants were smokers, while smoking is one of the significant factors influencing SAF measurements. A recent review summarised published reference data in various populations. It was apparent that accurate reference data in otherwise healthy individuals are lacking (35). The wealth of SAF data obtained from participants in the Lifelines Cohort Study poses an excellent opportunity to re-establish reference values in healthy individuals. The present study aims to establish better and more robust reference data as a strong basis for future studies and to facilitate proper evaluation of increased SAF measurements in various pathologic conditions.

## 2 Materials and methods

### 2.1 Study population

This study utilises data obtained from participants of the Lifelines Cohort Study (Lifelines, https://www.lifelines.nl), an extensive population-based survey in the northern region of the Netherlands. Lifelines is a multidisciplinary prospective population-based cohort study examining the health and health-related behaviours of 152,180 adult persons in a unique three-generation design. It employs a broad range of investigative procedures in assessing the biomedical, sociodemographic, behavioural, physical, and psychological factors contributing to the health and disease of the general population, with a particular focus on multi-morbidity. The design of the Lifelines Study has been described previously (36, 37).

Lifelines’ recruitment strategy has been reported previously (38). In summary, the recruitment aimed to include three generations of participants. In the first stage, all GPs in the three northern provinces of the Netherlands were invited to participate and asked to invite their registered patients aged 25–49 years. Patients who were unable to read Dutch or who had limited life expectancy due to severe illness were excluded by the GP and not invited to participate. Individuals who gave written informed consent were included as the “index population.” Subsequently, all persons in the index population were asked to indicate whether family members (partner, parents, parents-in-law, and children) could be invited to participate, and to provide their contact details. Those who gave their informed consent were included in the study as “family member.” Furthermore, persons 18 years and older could participate in this study through “self-registration” via the Lifelines website. These self-registrants were also asked to invite family members as outlined above. Individuals who had no family member participating in the study were not excluded.

For the present study, we evaluated the records of all 82,904 Lifelines participants in whom validated SAF measurements were available. Participants were eligible if they were of Western European descent, between 18 and 90 years of age, and underwent baseline investigations with a validated SAF measurement between 2007 and 2013. We did not include participants with extreme values of SAF (<0.8: *n* = 6 or >4.5: *n* = 28) as these likely represent measurement errors. **Supplementary Figure 1** shows the deposition of the participants. **Supplementary Table 1** briefly describes the baseline characteristics of the participants with and without validated SAF measurement.

### 2.2 Clinical examination

With the aid of self-administered questionnaires, information was collected at the baseline examination about health behaviour and past and current diseases, including alcohol intake and smoking habits [never, former, and current smoking, as well as time since smoking cessation (in former smokers) and pack-years of cigarette smoking]. For the assessment of physical activity, the Lifelines Physical Activity Score (LLPAS) was used. The LLPAS was based on the Short Questionnaire to Assess Health-Enhancing Physical Activity (SQUASH) (39) and indicated the number of minutes per week of moderate-to-vigorous physical activity (MVPA) during sports, leisure time, and active commuting (40). A certified research assistant performed anthropometric measurements and verified a participant’s medication use (36, 37). Body mass index (BMI) was calculated as weight/height². Medication use was scored according to the Anatomical Therapeutic Chemical Classification System.

### 2.3 Skin autofluorescence

SAF was measured non-invasively using a calibrated AGE Reader™ (Diagnoptics Technologies, Groningen, the Netherlands). The AGE Reader contains a light source, emitting light with a wavelength between 300 and 420 nm. While the volar side of the arm rests on the AGE Reader, only the emitted and reflected light is measured by a spectrometer in the 300–600-nm range. SAF is calculated as the ratio of emitted light (420–600 nm) and excitation light (300–420 nm), expressed as arbitrary units (AU). The mean value of three validated subsequent SAF measurements performed within 5 min is used for all analyses.

### 2.4 Biochemical measurements

Blood samples were taken between 08:00 and 10:00 a.m. after an overnight fast. Routine laboratory measurements were performed on the same day. Haematological parameters were measured on an automated Sysmex Haematology Analyser (Sysmex Corporation, Kobe, Japan). Serum creatinine was measured on a Roche Modular P chemistry analyser (Roche, Basel, Switzerland), and renal function was calculated as estimated (e)GFR with the 2009 formula developed by the Chronic Kidney Disease Epidemiology Collaboration (41). Total cholesterol and HDL-cholesterol were measured using an enzymatic colorimetric method, triacylglycerol (TG) using a colorimetric UV method, and LDL-cholesterol using an enzymatic method, on a Roche Modular P chemistry analyser (Roche). Fasting blood glucose was measured using the hexokinase method. Glycated haemoglobin (HbA_1c_, EDTA-anticoagulated) was analysed using an NGSP-certified turbidimetric inhibition immunoassay on a Cobas Integra 800 CTS analyser (Roche Diagnostics Nederland, Almere, the Netherlands).

### 2.5 Definitions and statistical analyses

Type 2 diabetes (T2D) was either self-reported or ascertained based on the use of oral blood-glucose-lowering medication or insulin use started >1 year after diabetes diagnosis, or a new diabetes diagnosis. The latter was defined as elevated fasting blood glucose ≥7.0 mmol/L or HbA_1c_ ≥6.5% at the baseline Lifelines screening. Type 1 diabetes was defined as any type of diabetes where insulin therapy was started within 1 year of diagnosis. Metabolic syndrome (MetS) was defined according to the revised NCEP ATPIII (R-ATPIII); at least three of the five metabolic risk components must be present to diagnose MetS. These metabolic risk components include 1) systolic blood pressure ≥130 mmHg and/or diastolic blood pressure ≥85 mmHg and/or use of antihypertensive drugs; 2) fasting blood glucose ≥5.6 mmol/L and/or use of blood glucose-lowering medication and/or diagnosis of T2D; 3) HDL-cholesterol levels <1.03 mmol/L in men and <1.30 mmol/L in women and/or use of lipid-lowering medication influencing these parameters; 4) triglyceride levels ≥1.70 mmol/L and/or use of triglyceride-lowering medication; and 5) waist circumference ≥102 cm in men and ≥88 cm in women (42, 43). Cardiovascular disease was defined as present when participants reported any one of the following diseases or events: myocardial infarction, percutaneous transluminal coronary angioplasty (PTCA), stent positioning, or coronary artery bypass grafting (CABG), transient ischaemic attack (TIA) and cerebrovascular accident (CVA), intermittent claudication, or peripheral artery vascular surgery.

A participant was considered healthy when he or she did not use any medication, had no earlier diagnosis of diabetes or CVD, had normal fasting blood glucose and HbA_1c_, and an eGFR above 60 mL/min/1.73 m^2^. Based on smoking habits, we separated our analyses between never, former, and current smokers.

Previously, we have shown that individuals with metabolic syndrome had higher SAF (19), and, in addition, that there was a positive association between BMI and SAF (18). However, the latter relationship was more complex and non-linear (**Supplementary Figure 2**). Therefore, reference values for SAF were established in healthy participants who had never smoked, had a BMI below 35 kg/m^2^, and did not have metabolic syndrome. As a sensitivity analysis, we recalculated reference values by only including never-smoking participants with a BMI between 20 and 30 kg/m^2^. Linear regression was performed between the SAF score and age to obtain the regression slope and to calculate age-adjusted SAF *Z*-scores separately by sex. SAF reference values were calculated for each age decade (18–30, 31–40, 41–50, 51–60, 61–70 years). Although the dataset contains several participants older than 70 years, we did not use these data to calculate reference values because of a high possibility of both participation and survival bias, as has been demonstrated in large population-based studies like the UK Biobank (44).

To evaluate deviation from the reference values in healthy never-smoking individuals, we calculated and plotted decade-specific mean SAF values for the following groups of participants:

1. former and current smokers;
2. participants with metabolic syndrome;
3. participants with type 2 diabetes, stratified for smoking status, and the absence or presence of clinical CVD;
4. participants with clinical CVD, stratified for smoking status, and with or without diabetes; and
5. participants with renal impairment, defined as eGFR below 60 mL/min/1.73 m^2^. In addition, we assessed the degree of deviation from the reference values based on calculated pack-years of smoking in current smokers.

To evaluate the association between regular physical activity and SAF scores, subgroups of physical activity were defined as follows: 0, 1–149, 150–299, 300–599, and 600+ min/week moderate-to-vigorous physical activity, similar to a recent work by Marks-Vieveen et al. (45). The association between physical activity and age-adjusted SAF was calculated by ANOVA, unadjusted and adjusted for sex, pack-years of smoking (in former and current smokers), and presence of MetS (for all healthy subgroups). The non-linear relationship between moderate-to-vigorous physical activity and age-adjusted SAF scores was evaluated further with restricted cubic spline curve (RCS) regression using the Stata package postrcspline (46).

All analyses were performed in PASW Statistics (Version 28, IBM, Armonk, NY, USA) and Stata Statistical Software (Stata MP version 18.1; Stata Corp, College Station, TX, USA). Missing data were not imputed. Data are presented as means ± SD, or median and interquartile range (IQR) when not normally distributed. Means were compared between relevant groups with ANOVA. The Wilcoxon rank sum test compared variables between groups when they were not normally distributed. The chi-square test was used to analyse categorical variables. Values of *p* < 0.05 were considered statistically significant. Privacy guidelines of the Lifelines Cohort Study do not permit publication of data with *N* < 10 observations.

## 3 Results

### 3.1 Demographics

A total of 82,870 participants had a valid SAF measurement at the baseline Lifelines screening. **Table 1** shows the characteristics of the healthy participants according to their baseline smoking status. In never-smokers, the mean ( ± SD) age was 41.4 ± 11.0 years, and BMI was 25.4 ± 3.9 kg/m^2^. By definition, they had normal concentrations of fasting glucose and HbA_1c_ and normal eGFR, while 8.1% fulfilled the criteria for metabolic syndrome.

**Table 1:** Clinical characteristics of healthy participants according to smoking status.

Characteristic	Healthy never-smokers (*n* = 17,971)	Healthy former smokers (*n* = 11,474)	Healthy current smokers (*n* = 8,413)	*p*
Sex (*n*; male/female)	9,394/8,577 (52.3/47.7%)	5,554/5,920 (48.4/51.6)	4,805/3,608 (57.1/42.9%)	<0.001
Age (years)	41.4 ± 11.0	47.0 ± 10.6	40.5 ± 10.4	<0.001
BMI (kg/m^2^)	25.4 ± 3.9	26.0 ± 3.8	25.5 ± 3.8	<0.001
Waist circumference (cm)	89 ± 11	91 ± 11	90 ± 11	<0.001
Glucose (mmol/L)	4.9 ± 0.5	5.0 ± 0.5	4.9 ± 0.5	<0.001
HbA_1c_ (mmol/mol)	36 ± 3	37 ± 3	37 ± 3	<0.001
eGFR (mL/min/1.73 m^2^)	99 ± 14	95 ± 13	102 ± 13	<0.001
KDIGO stages G1/G2 (%)	73.5/26.5	65.3/34.7	81.4/18.6	<0.001
Pack-years of smoking (*n*)	N.A.	6.0 (2.6–12)	12.0 (6.0–19.5)	<0.001
Presence of obesity* (%)	15.0	13.3	11.4	<0.001
Presence of metabolic syndrome (%)	8.1	10.4	13.3	<0.001
SAF (AU)	1.80 ± 0.36	1.95 ± 0.39	1.97 ± 0.45	<0.001
Inclusion mode (%)				<0.001
Family doctor	63.2	56.7	67.2	
Included family member	26.2	29.4	23.4	
Self-registered	10.6	13.9	9.3	

Data are presented as numbers, means ± SD, median (IQR) or percentages. BMI, body mass index; CVD, cardiovascular disease; eGFR, estimated glomerular filtration rate; HbA_1c_, glycated haemoglobin; KDIGO, Kidney Disease: Improving Global Outcomes; G1, eGFR normal or high ≥90; G2, eGFR mildly decreased 60–89 mL/min/1.73 m^2^; SAF, skin autofluorescence; N.A., not applicable.

*BMI > 30 kg/m^2^.

### 3.2 Reference values in healthy never-smokers

As mentioned above, there was a significant non-linear association between BMI and SAF in healthy never-smokers (**Supplementary Figure 2**), with SAF being significantly higher in participants with a BMI of 35.0 kg/m^2^ and higher. Similarly, age-adjusted SAF scores were significantly higher (+0.10, *p* = 0.032) in female healthy never-smokers with MetS vs. those without MetS, but not in male healthy never-smokers. We established the final reference values for healthy individuals in 8,179 men and 7,930 women between 18 and 70 years, who had never smoked, had a BMI below 35 kg/m^2^, and did not meet the MetS criteria. Mean SAF linearly increased with age, and the linear regression analyses yielded the following prediction for SAF, separately by sex:


Males: SAF predicted = (0.0191 × age) + 1.038

Females: SAF predicted = (0.0188 × age) + 0.994

**Figure 1:**
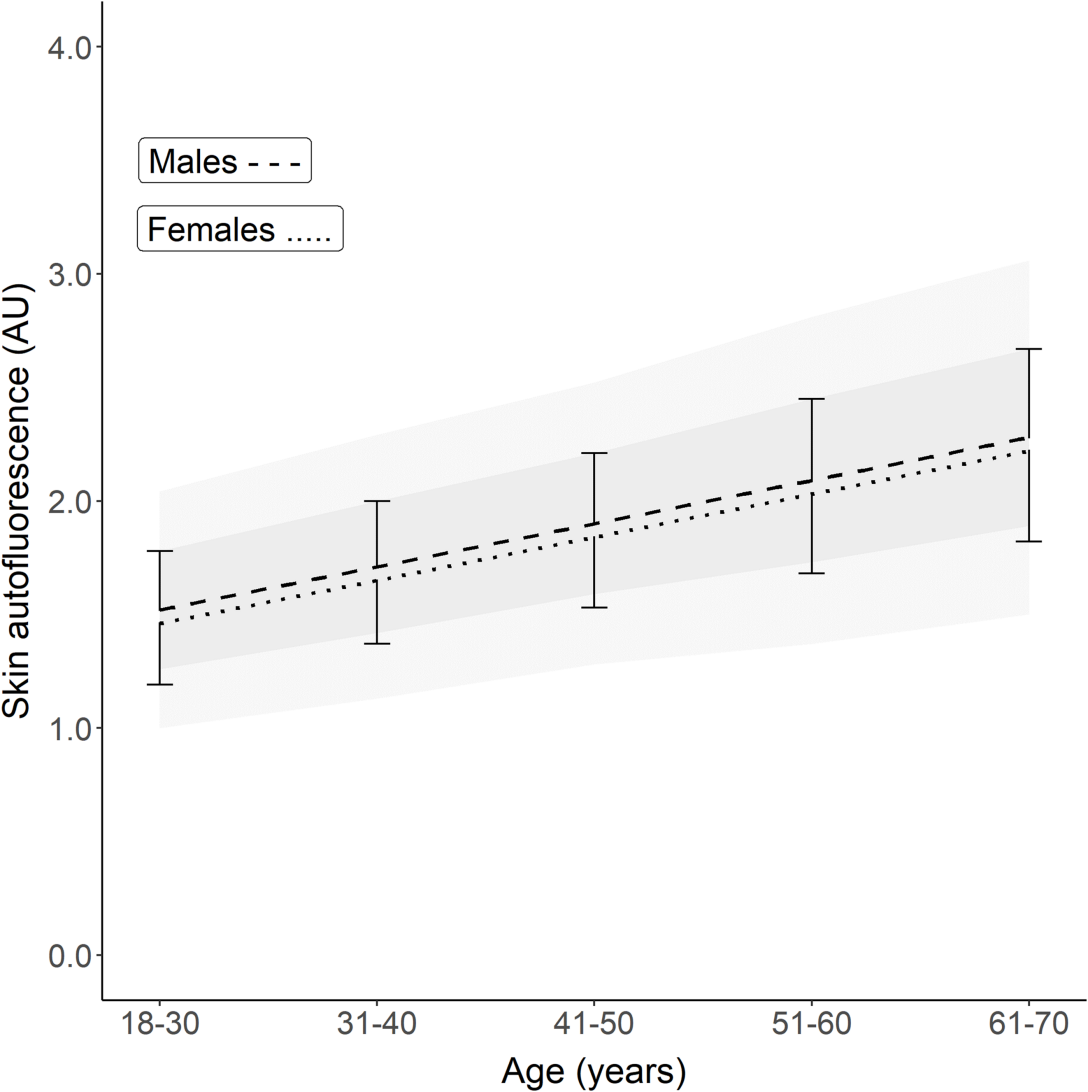
Reference values of skin autofluorescence for healthy Lifelines participants who never smoked, had a BMI <35 kg/m^2^, and no metabolic syndrome. The graph shows the mean ± SD for each age decade based on the linear regression equation. Male reference values are depicted as mean ± SD (darker grey area) and ± 2SD (lighter grey area). On average, men have a 0.04–0.05 higher SAF value for each decade (*p* = 0.016). The regression equations are: Males: SAF predicted = (0.0191 × age) + 1.038. Females: SAF predicted = (0.0188 × age) + 0.994.

These regression lines are significantly different, with *p* = 0.016. **Table 2** and **Figure 1** show the calculated SAF reference values for each age decade, separated by sex. For all age decades, SAF values were significantly higher in men than in women.

**Table 2:** Reference values for skin autofluorescence in healthy individuals aged 18–70 years.

Age (years)	Males		Females	
	SAF	*N*	SAF	*N*
18–30	1.52 ± 0.26	1,800	1.46 ± 0.27	1,120
31–40	1.71 ± 0.29*	2,297	1.65 ± 0.28*	2,273
41–50	1.90 ± 0.31*	3,042	1.84 ± 0.31*	3,186
51–60	2.09 ± 0.36*	713	2.03 ± 0.35*	862
61–70	2.28 ± 0.39*	327	2.22 ± 0.40*	489

Data show SAF values as mean ± SD for men and women separately and the number of individuals per decade group used for the linear regression analysis.

*p < 0.001 vs. previous age decade.

The sensitivity analysis of the association between age and SAF, in participants who had never smoked, did not meet the MetS criteria, and had a BMI between 20 and 30 kg/m^2^, yielded similar regression equations:


Males: SAF predicted = (0.0191 × age) + 1.037

Females: SAF predicted = (0.0187 × age) + 0.998

### 3.3 Smoking status

**Figure 2:**
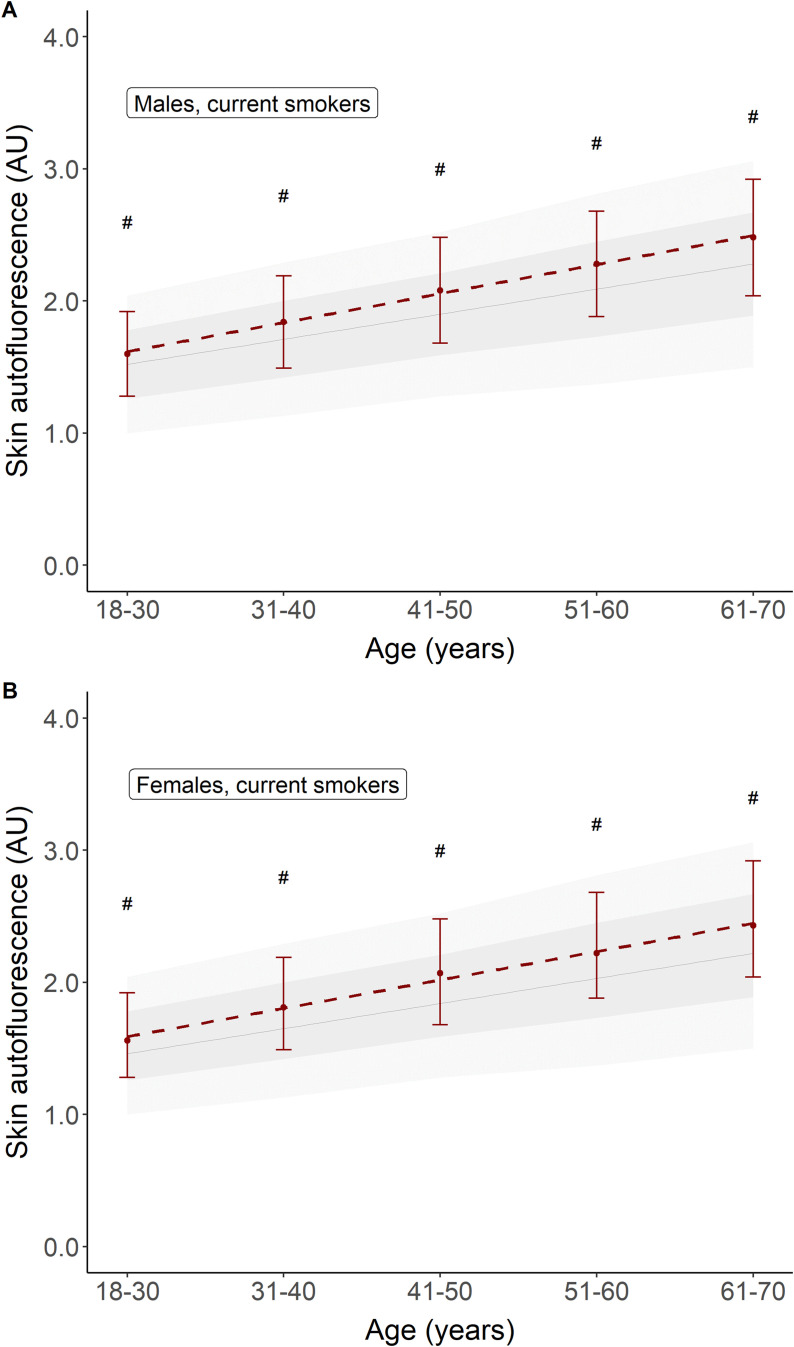
Comparison of mean skin autofluorescence for healthy participants currently smoking (in red) vs. healthy participants who never smoked (e.g., reference values). Panel **(A)** shows males, panel **(B)** shows females. Reference values are depicted as mean ± SD (darker grey area) and mean ± 2SD (lighter grey area). #p < 0.001 vs. reference values.

**Figure 3:**
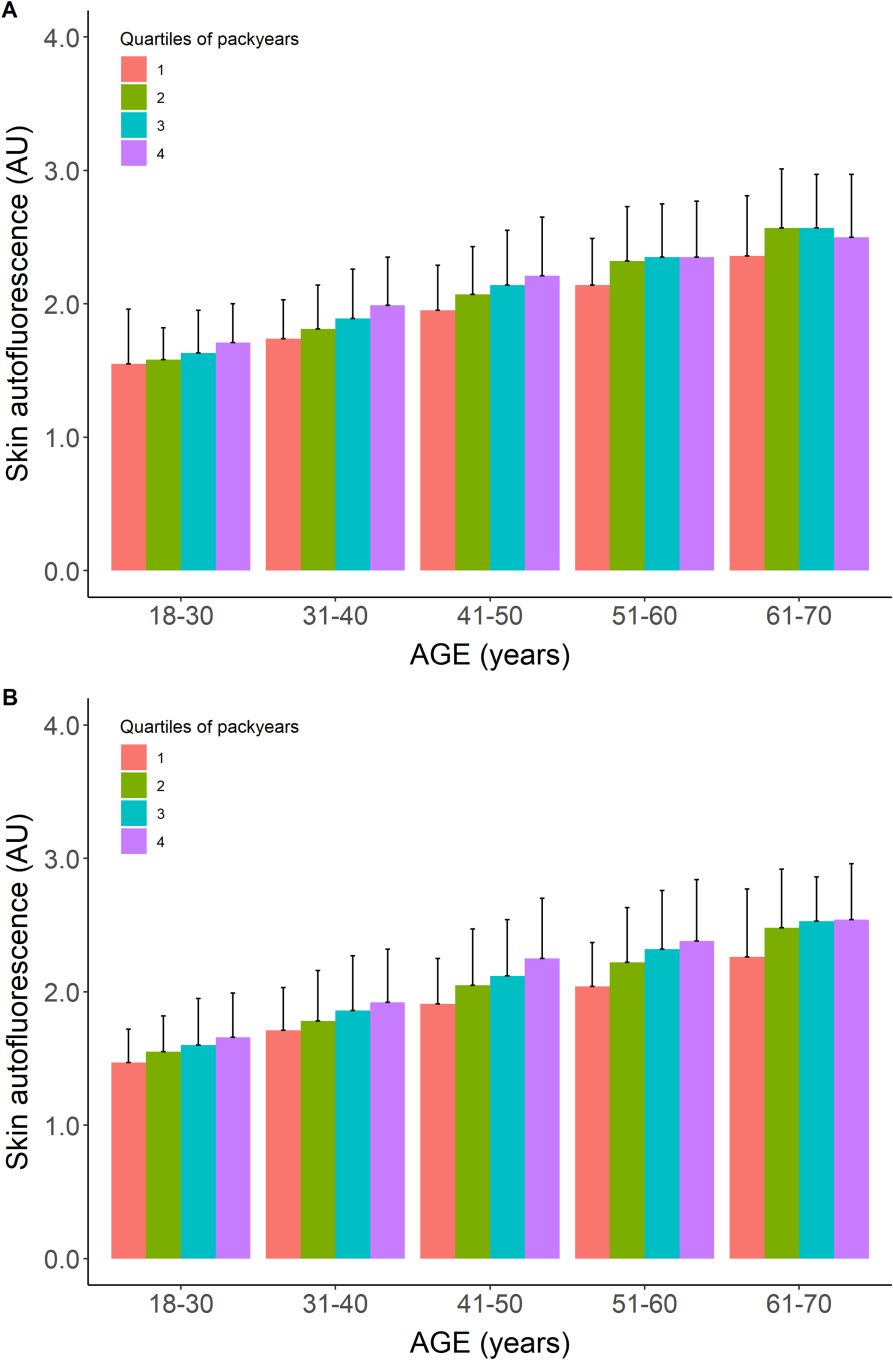
Mean skin autofluorescence values for healthy current smokers stratified for quartiles of pack-years of cigarette smoking. Panel **(A)** show males, panel **(B)** shows females. Data shown as means ± SEM. For each age decade, the mean value of SAF increases with a higher number of pack-years.

**Table 1** shows the baseline characteristics for participants who were considered healthy but were former or current smokers. Former smokers were older (47.0 ± 10.6 years) and had a higher BMI, a 10.4% prevalence of the metabolic syndrome, and a median of 6 pack-years of smoking. Current smokers had the highest prevalence of metabolic syndrome and a median of 12 pack-years of smoking, while other baseline characteristics were comparable to those of the never-smokers. Mean age- and sex-adjusted SAF scores were the highest in current smokers. Their mean SAF score was 1.97 ± 0.45 AU, with a mean age-adjusted *Z*-score of 0.5 SD above that of healthy never-smokers. **Figure 2** shows their mean SAF values per age decade. Although all mean values for current smokers fall within 1 SD of the reference interval of healthy never-smoking individuals, it is clear that the regression line for current smokers progressively deviates from the regression line for never-smokers. One of the primary reasons for this is the cumulative effect of smoking. Indeed, when we plot pack-years of smoking per decade, we can observe a clear trend of higher mean SAF with a higher number of pack-years of smoking in each age decade, in both sexes (**Figure 3**).

### 3.4 Disease states

**Figure 4:**
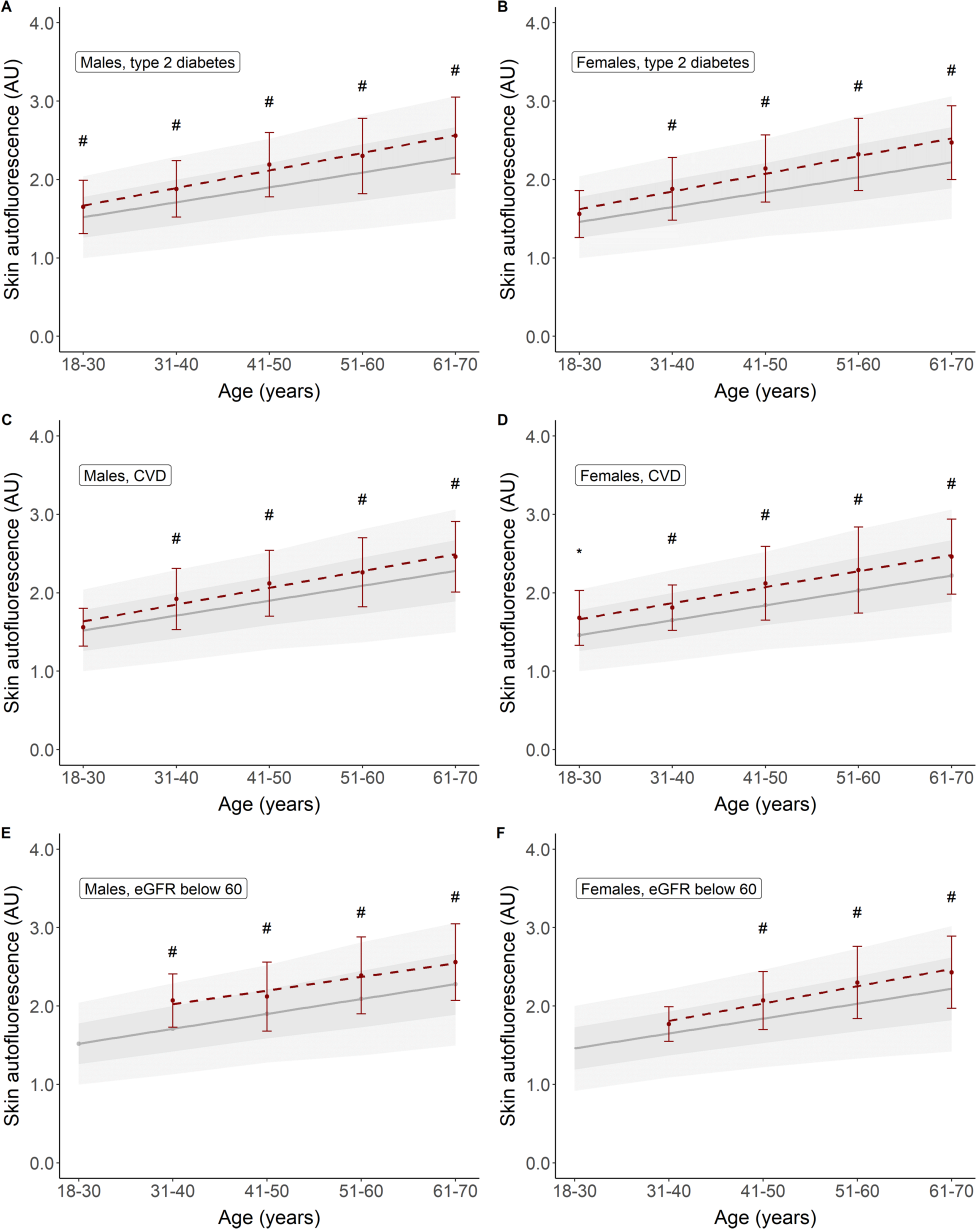
Skin autofluorescence for individuals with type 2 diabetes **(A, B)**, CVD **(C, D)**, and reduced eGFR **(E, F)**. Reference values depicted as mean ± SD (darker grey area) and mean ± 2SD (lighter grey area). #*p* < 0.001, *p < 0.01 vs. reference values.

**Table 3** shows a brief overview of the baseline characteristics of the participants with type 2 diabetes, CVD, and renal impairment. Their mean age was considerably higher than that of healthy participants, with higher BMI and glycaemic variables in the participants with type 2 diabetes and a high percentage (60%–75%) of combined former and current smoking. In **Figure 4**, we show the mean SAF values for individuals with type 2 diabetes, those with CVD, and those with reduced eGFR, separately by sex. For almost each decade, both people with type 2 diabetes, CVD, and renal impairment have significantly higher SAF values compared to healthy individuals.

**Table 3:** Clinical characteristics of disease participants.

Characteristic	Type 2 diabetes (*n* = 2,571)	CVD (*n* = 1,953)	Renal impairment (*n* = 825)	*p*
Sex (*n*; male/female; %)	1,338/1,233 (52.0/48.0)	1,288/675 (65.6/34.4)	352/473 (42.7/57.3)	<0.001
Age (years)	56.7 ± 11.7	58.5 ± 12.2	65.5 ± 11.4	<0.001
BMI (kg/m^2^)	30.3 ± 5.4	27.9 ± 4.2	27.9 ± 4.2	<0.001
Waist circumference (cm)	104 ± 14	98 ± 12	97 ± 12	<0.001
Glucose (mmol/L)	7.6 ± 2.2	5.5 ± 1.2	5.5 ± 1.1	<0.001
HbA_1c_ (mmol/mol)	52 ± 12	41 ± 17	41 ± 17	<0.001
eGFR (mL/min)	91 (79–102)	87 (75–97)	55 (50–58)	<0.001
Current smoking (%)	18.9	20.4	10.6	<0.001
Former smoking (%)	47.6	55.0	48.7	<0.001
Pack-years of smoking (*n*)	6 (0–20)	11 (0–24)	1 (0–13)	<0.001
Presence of obesity* (%)	46.4	26.5	25.3	<0.001
Presence of metabolic syndrome (%)	68.2	35.9	37.0	<0.001
Presence of type 2 diabetes (%)	100	15.7	15.4	ND
Presence of CVD (%)	12.0	100	17.1	ND
SAF (AU)	2.34 ± 0.52	2.32 ± 0.52	2.47 ± 0.53	<0.001
Inclusion mode (%)				<0.001
Family doctor	35.3	30.0	14.2	
Included family member	50.7	56.6	70.9	
Self-registered	14.0	13.4	14.9	

Data are presented as numbers, means ± SD, median (IQR), or percentages.

BMI, body mass index; CVD, cardiovascular disease; eGFR, estimated glomerular filtration rate; HbA_1c_, glycated haemoglobin; SAF, skin autofluorescence; ND, not determined.

*BMI > 30 kg/m^2^.

**Figure 5:**
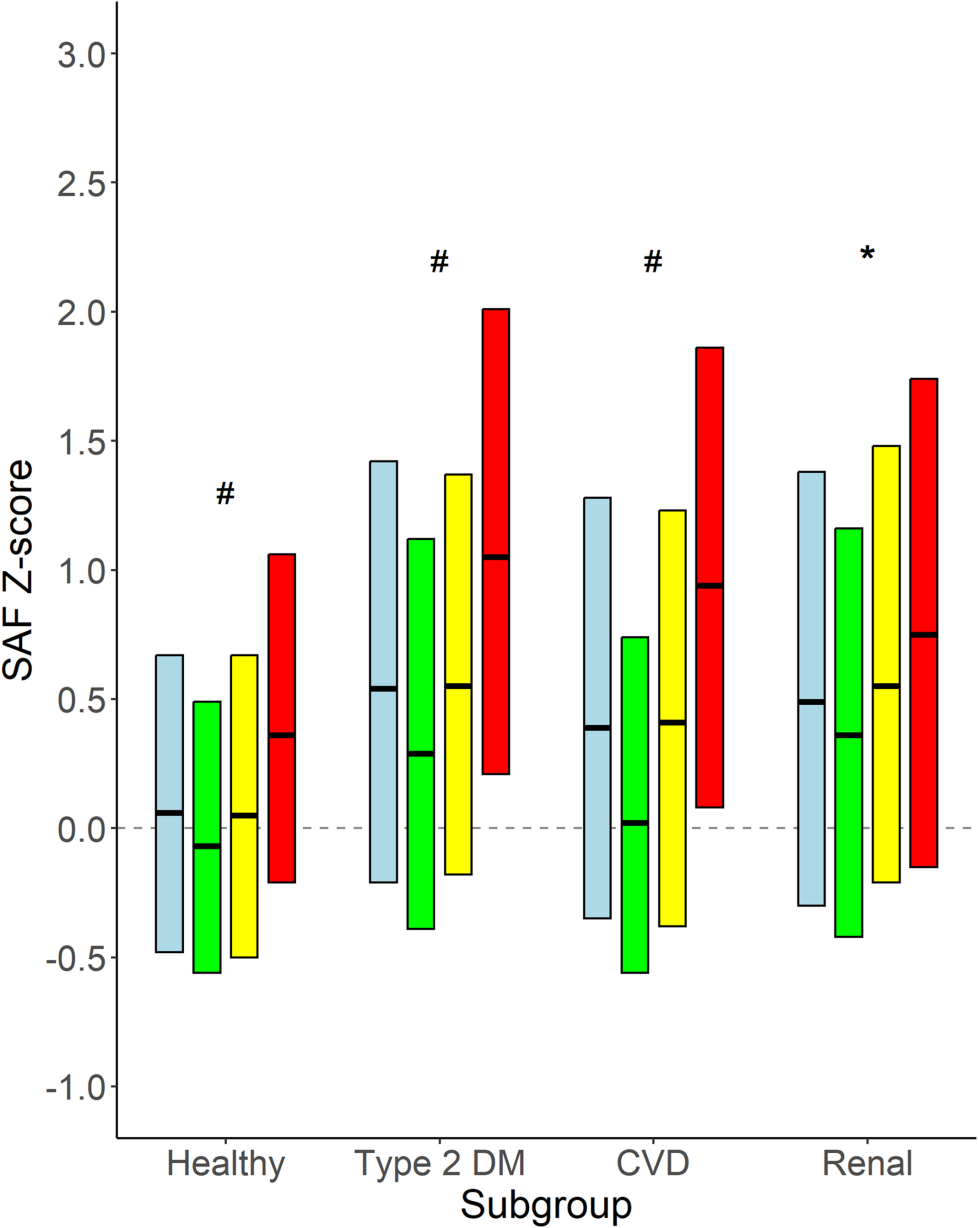
Age-adjusted SAF score for healthy individuals and participants with type 2 diabetes, CVD, and renal impairment. Data shown as median and 25th and 75th percentiles for each category of participants, e.g., healthy participants and participants with type 2 diabetes, CVD, and renal impairment, respectively, stratified for smoking status. Renal impairment was defined as eGFR <60 mL/min/1.73 m^2^. Colours indicate subgroups: Light blue: all participants in the subgroup; Green: never smokers; Yellow: former smokers; Red: current smokers. #p < 0.001 never vs. former and vs. current smokers, former vs. current smokers. *p < 0.01 never vs. former and vs. current smokers.

**Figure 5** shows the mean age-adjusted SAF values for the different groups of disease states, stratified for smoking status. It becomes apparent that in all groups of participants, e.g., healthy individuals, people with type 2 diabetes, and those with CVD or renal impairment, SAF values for current smokers are higher than in former smokers and never-smokers. Current smokers with type 2 diabetes and current smokers with CVD have a median SAF *Z*-score of 1.0 SD, which is above the SAF score for healthy individuals.

**Figure 6:**
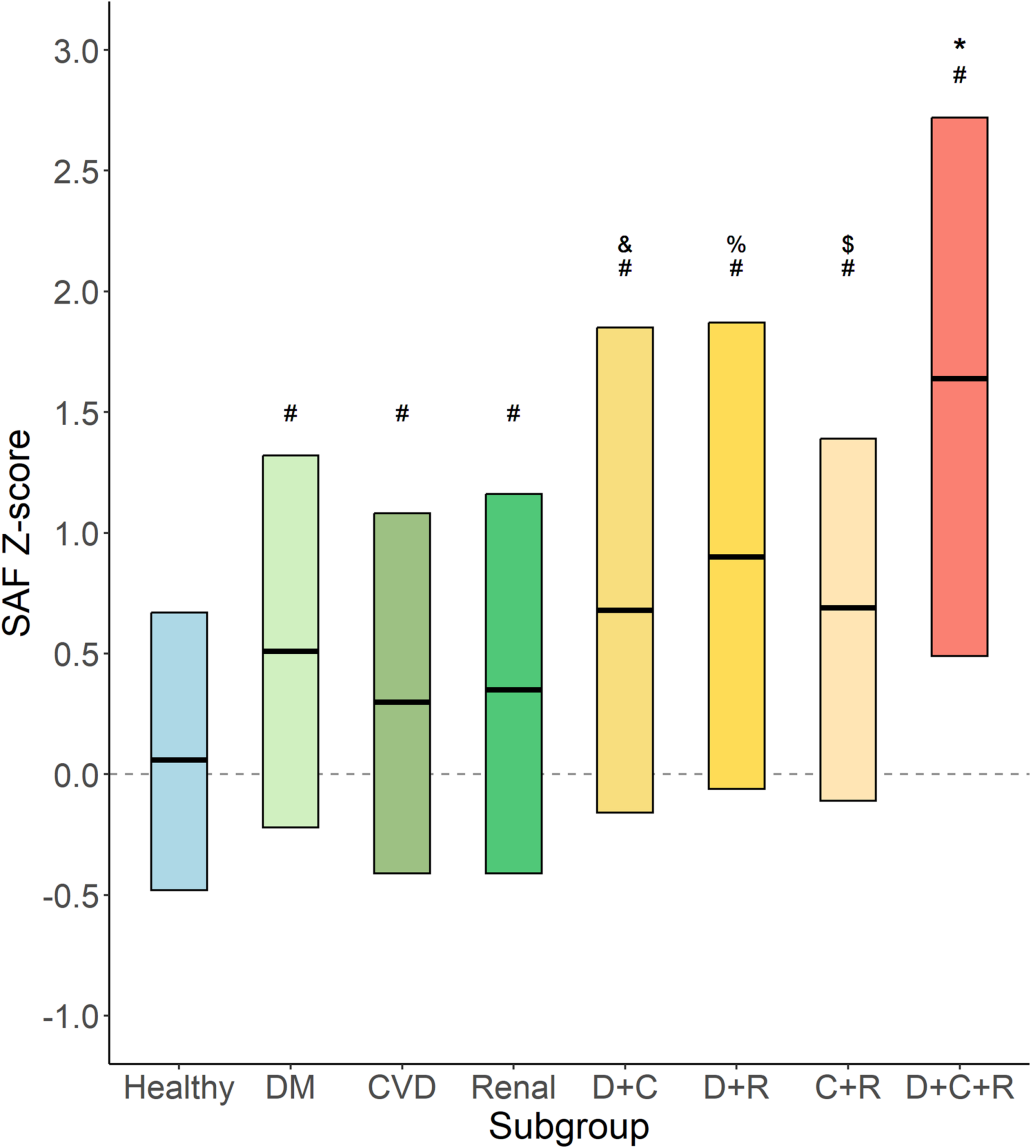
Age-adjusted SAF score for specific disease groups. Data shown as median and 25th and 75th percentiles for each category of participants, e.g., healthy participants (Healthy, *n* = 37,917); type 2 diabetes (DM, *n* = 2,172) without CVD and with normal renal function; cardiovascular disease (CVD, *n* = 1,552) without diabetes and with normal renal function; renal impairment (Renal, n = 595) without diabetes and without CVD; type 2 diabetes and CVD (D+C, *n* = 270) with normal renal function; type 2 diabetes and renal impairment (D+R, *n* = 88) without CVD; CVD and renal impairment (C+R, *n* = 102) without diabetes; and type 2 diabetes, CVD, and renal impairment (D+C+R, *n* = 39). #*p* < 0.001 vs. healthy. &*p* < 0.005 vs. DM and vs. CVD. %*p* < 0.005 vs. DM and vs. renal. $*p* < 0.05 vs. CVD. *p < 0.005 vs. D+C and vs. C+R.

**Figure 6** shows the age-adjusted SAF scores for specific disease groups, alone and in each combination. From the data, it becomes apparent that all disease groups have SAF *Z*-scores higher than observed in healthy individuals. The relatively small subgroup of participants with type 2 diabetes, CVD, and renal impairment (*n* = 39) has the highest age-adjusted SAF values (*p* < 0.005 vs. diabetes plus CVD and vs. CVD plus renal impairment).

### 3.5 Physical activity

**Figure 7:**
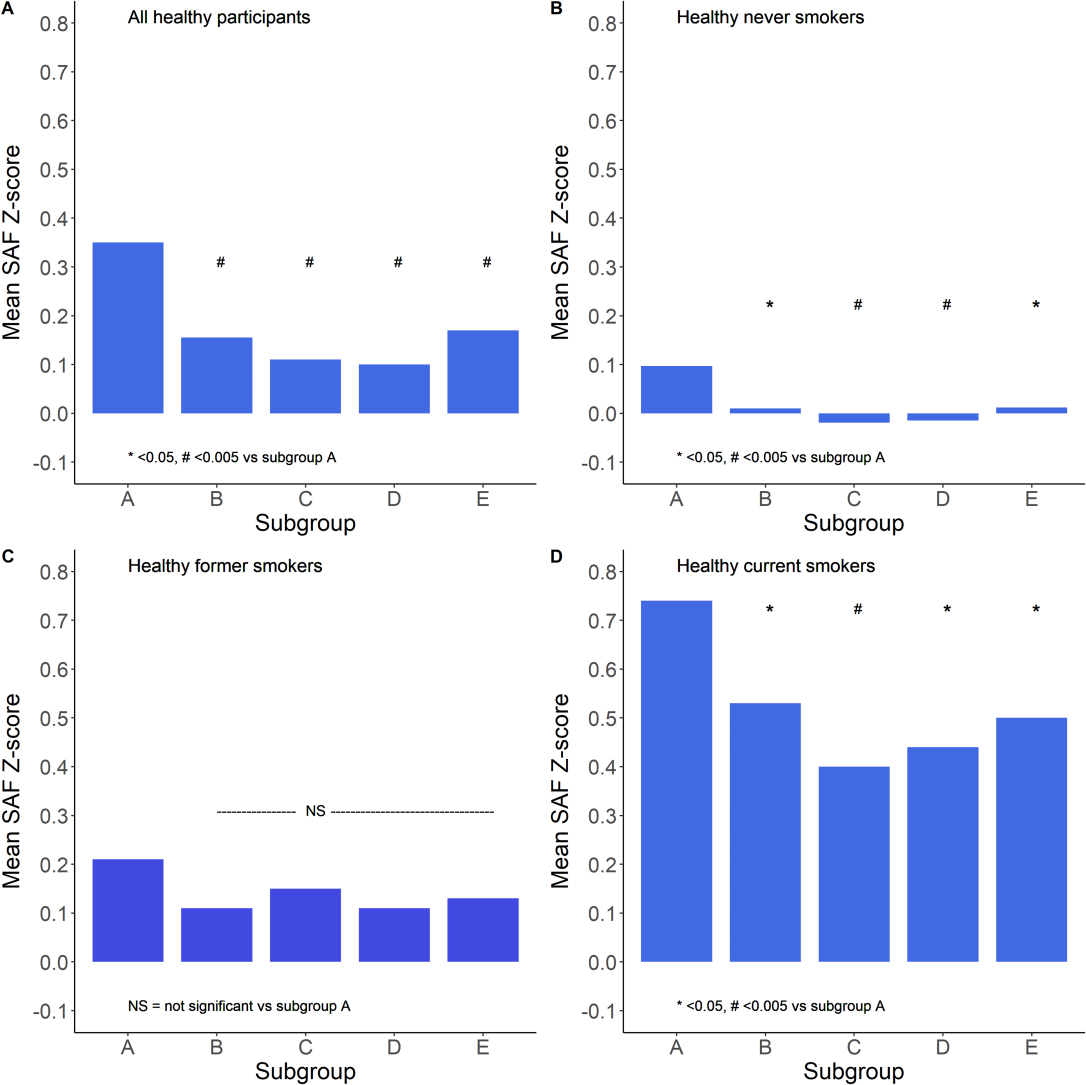
Relationship between the extent of moderate–vigorous physical activity and mean SAF Z-scores. Subgroups are defined as A: 0, B: 1–149, C: 150–299, D: 300–599, and E: 600+ min/week moderate-to-vigorous physical activity. Panel **(A)** shows all healthy participants, panel **(B)** shows healthy never smokers, panel **(C)** shows healthy former smokers, and panel **(D)** shows healthy current smokers. *p < 0.05, #p < 0.005 vs. subgroup A (sedentary group), tested by ANOVA with Tukey correction for multiple comparisons.

Data regarding physical activity (PA) were available in 34,516 healthy participants, 16,362 never-smokers, 10,489 former smokers, and 7,665 current smokers. **Figure 7** shows the mean SAF scores for the five physical activity groups. It is apparent that there was a complex curvilinear relationship between PA and age-adjusted SAF score. In healthy never-smokers, the mean SAF was the highest in sedentary participants, e.g., 0 min/week moderate-to-vigorous physical activity. SAF scores were not significantly different between participants with higher levels of physical activity. In healthy former smokers, the SAF score was the highest in sedentary individuals, but both unadjusted and adjusted for pack-years of smoking and presence of the metabolic syndrome, there was no statistically significant difference between the five subgroups. In healthy current smokers, the mean SAF was the highest in sedentary participants, and it was significantly higher (*p* < 0.001) than in the other four subgroups; however, this was also the subgroup with the highest past cigarette consumption. When adjusted for pack-years of smoking and presence of MetS, the difference between subgroups 1 and 2 (e.g., sedentary participants vs. those with low PA) was not significant (*p* = 0.052).

**Figure 8:**
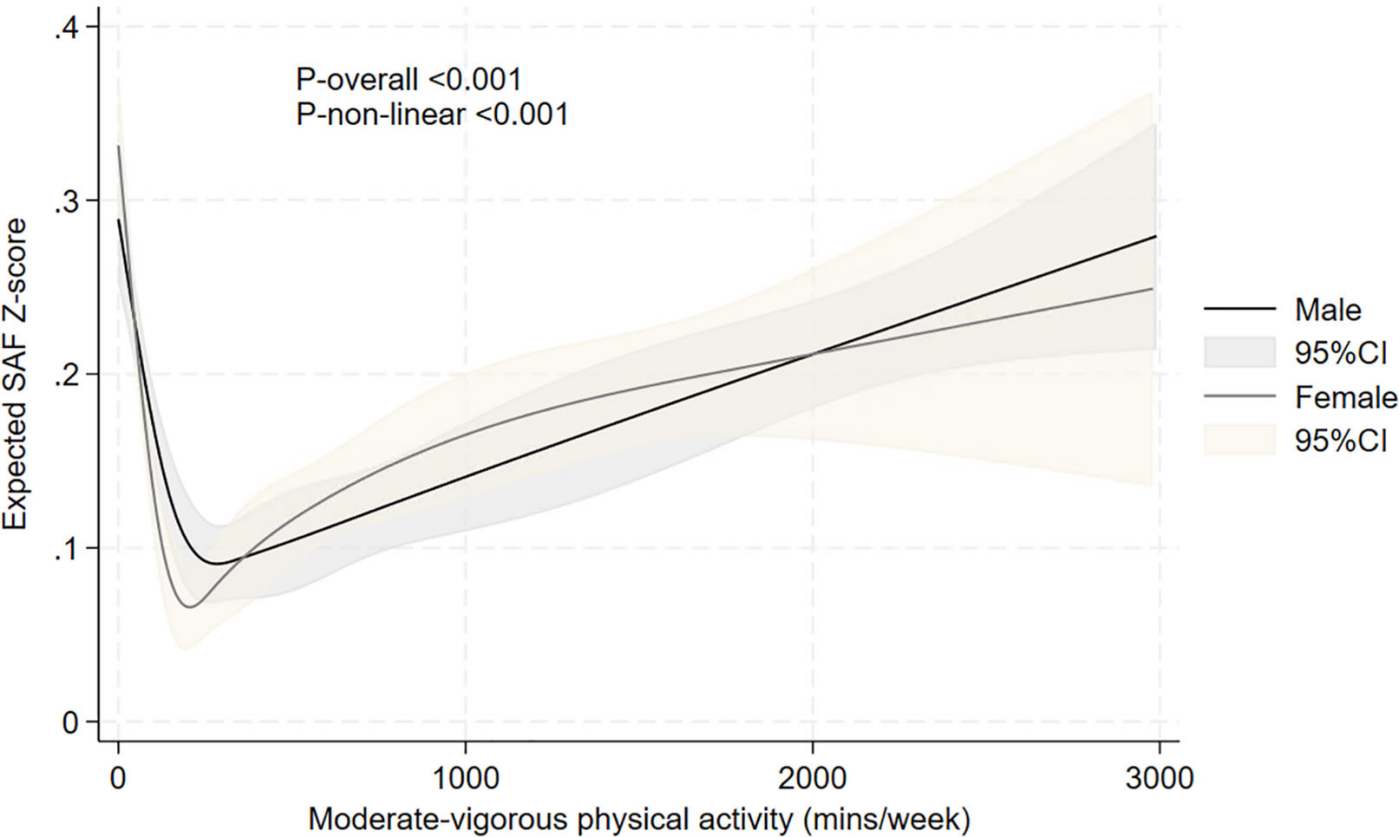
Relationship between moderate-to-vigorous intensity physical activity and expected age- and sex-adjusted SAF scores, evaluated using restricted cubic splines. The results are presented using solid black and grey lines for the expected SAF *Z*-score in men and women, respectively, and grey and light-brown shadow (indicating the 95% confidence interval) for the respective confidence intervals. *p*-values for non-linearity and overall association are also presented.

**Figure 8** summarises the restricted cubic spline analyses, showing a U-shaped relationship between moderate-to-vigorous intensity physical activity (in min/week) and age- and sex-adjusted SAF scores. The lowest mean SAF score was around the 200-min/week physical activity level. Higher durations of moderate-to-vigorous physical activity were associated with a gradual increase in SAF scores.

## 4 Discussion

The current study reflects the evaluation of reference values for skin autofluorescence in a large group of healthy Dutch individuals who never smoked. We have shown a gradual increase of SAF with increasing age in a linear manner, while SAF is systematically higher in men than in women, in current smokers vs. former and never-smokers, and in people with type 2 diabetes, clinical CVD, or impaired renal function. Also, SAF increases further with a higher consumption of cigarettes, defined as pack-years of smoking.

Typically, reference values for SAF in previous studies have been based on small-sized studies (34, 35). When we compare our current dataset with a previous reference value set by Koetsier et al. (34), they reported in 332 healthy non-smokers that the predicted SAF could be calculated as 0.023 * age + 0.83. This will result in relatively lower SAF values in people under 50 years and higher SAF values in those over 50 years. Furthermore, they did not find sex differences in SAF. In one of our earlier papers, we presented age-adjusted SAF scores based on values obtained in a clinically healthy population, e.g., those free of diabetes and CVD (25). Here, we obtained higher normal SAF values per age decade because the general population was a mix of never, former, and current smokers. Our present data clearly show the effects of smoking on SAF, with SAF values being at least 0.5 AU (1 SD) higher in current smokers compared to never-smokers. Therefore, it is pivotal to use, for prognostication and clinical decision-making, reference data which have been obtained in a healthy population of participants who have never smoked. In their recent paper, Martinez-Garcia et al. gave an elegant overview of 10 studies in which cross-sectional reference data were reported (35). Only three of these studies based their reference data on non-smokers. A Dutch study included 32 women who were never-smokers, but this study was not aimed at establishing reference values, but to determine biological skin age (47). A study in Brazil reported the results of SAF measurements in 65 apparently healthy individuals aged between 20 and (approximately) 80 years, of whom none were current smokers, but details on previous smoking habits were not provided (48). Another study from Brazil described results obtained in 736 participants who were reported as non-smokers and who were without diabetes or chronic kidney disease (49). Again, previous smoking history was not reported. All other studies have based their reference data on groups of participants, which included never, former, and current smokers, with between 10% and 30% of participants considered as current smokers (28, 35). Such an evaluation will result in higher reference values and, as a consequence, underestimate the risk associated with increased SAF values. This becomes apparent when we compare our regression equation with earlier published ones, including the equation presented in the paper of Martinez-Garcia (35). Thus, the current study, which provides reference data obtained in almost 18,000 healthy individuals who had never smoked, can be regarded as an essential step in improving clinical decision-making and risk estimation based on skin autofluorescence measurements.

An essential aspect of establishing reference values, which has received little attention, is the possible effect of the use of specific medications on SAF. Indeed, a variety of drugs has been shown to influence the formation of advanced glycation end-products, by different mechanisms, and these drugs include angiotensin-converting enzyme (ACE) inhibitors, angiotensin receptor blockers (ARBs), statins, thiazolidinediones, aspirin, and metformin (50–53). For this reason, we defined being healthy as not using any type of medication. Although individual Lifelines participants may be using several of these drugs, assessing their possible effect on SAF scores is not feasible. First of all, because we do not have SAF measurements before and after the start of a specific medication, and second, analysing medication use in a cross-sectional evaluation may be influenced by indication bias: prescription of, for instance, statins and ACE inhibitors will be done in those with the highest cholesterol concentrations or blood pressure levels.

The current study and our previous study (25) show that SAF values in men are on average 0.05 AU higher than in women (**Table 2**), and this difference is consistent when assessing the full age range between 18 and 70 years. It should be repeated here that these results have been uniquely obtained in participants who have never smoked. Not all studies have reported sex differences. Some studies did not find differences, perhaps due to relatively low numbers of participants (e.g., below 1,000), hence very low power to establish or exclude differences (35), while other studies reported higher SAF in female participants. In a large study in Japan, comprising 10,946 participants (with 77% being female participants), no sex-specific analyses were reported (54). In a study from Slovakia with 1,385 participants, women had a mean 0.07 AU higher SAF than men, but this was only statistically significant for the age group 40–49 years (55). Those data do not allow adjustment for sex differences, for instance, in smoking habits (55).

Only a few earlier studies have reported an association between physical activity and SAF score. Isami used a straightforward definition of physical exercise and suggested that a higher physical activity score (ranging from “not at all” to “more than 4 times a week”) showed a linear association with SAF in 10,946 healthy Japanese adults. Our data clearly show that the relationship between physical activity and SAF is non-linear, which we could observe in both never-smokers and current smokers. The highest mean SAF score was observed in sedentary individuals, e.g., those reporting 0 min/week moderate-to-vigorous intensity physical activity. In contrast, those performing 150–299 min/week had the lowest mean SAF scores. Restricted cubic spline analysis revealed a clear U-shaped relationship between physical activity and mean SAF score, with a gradual increase of SAF scores with higher than 600 min/week moderate-to-vigorous physical activity (**Figure 8**). Indeed, although the health benefit of regular exercise has been clearly established, it has also been demonstrated that excessive exercise may result in increased production of reactive oxygen species (ROS) and, consequently, oxidative stress markers like ROS-induced end products of lipids, proteins, and various enzymatic and non-enzymatic antioxidants (56–58). This will add to the overall glycation and oxidation process in the body, which the AGE Reader detects (59). As has been reported earlier, AGEs on one hand modulate oxidative stress, and on the other hand, excessive oxidative stress accelerates the generation of AGEs (60, 61).

The strength of this evaluation is the unprecedentedly large number of participants whose data were available for analysis. This allowed for the separate assessment of the sex differences in SAF, the effects of current and previous smoking, the cumulative impact of pack-years of smoking, and the complex effects of physical activity. As such, the data from Lifelines have contributed to finding genes related to SAF (23, 24); the association of SAF with lifestyle (18) and cardiovascular risk factors (19, 22, 33); the association with individual components of the metabolic syndrome, including HDL-cholesterol and triglycerides (19); and the incidence of diabetes, CVD, and mortality (25, 26, 62, 63). As the Lifelines participants only underwent a baseline examination, the data can not be used to evaluate the possible effects of medications on SAF. Because of the Western European descent of the participants, the data can not be extrapolated to people from other ethnicities.

In conclusion, SAF increases linearly with age in healthy individuals. We provided robust reference values for SAF, established in healthy individuals of Western European descent, separately by sex, who have never smoked. Higher levels of SAF are observed in former and current smokers and in people with type 2 diabetes, CVD, and impaired renal function. The relationship between physical activity and SAF scores is complex, with higher SAF scores demonstrated in both sedentary people and those performing excessive amounts of moderate-to-vigorous physical exercise.

## Data Availability

The datasets presented in this article are not readily available because the article is based on data from the Lifelines Cohort Study. Lifelines adheres to standards for data availability, and allows access for reproducibility of the study results. The data catalogue of Lifelines is publicly accessible at www.lifelines.nl. The dataset supporting the conclusions of this article is available through the Lifelines organisation (e-mail: research@lifelines.nl). A fee is required for data access. Requests to access the datasets should be directed to https://www.lifelines.nl.
