# Supplementary figures and images for: Reference values and biological factors influencing skin autofluorescence
Source: Front Endocrinol (Lausanne). 2025 Nov 6;16:1700892. doi: 10.3389/fendo.2025.1700892 (PMC12631760; doi:10.3389/fendo.2025.1700892)

Supplemental Figure 1.

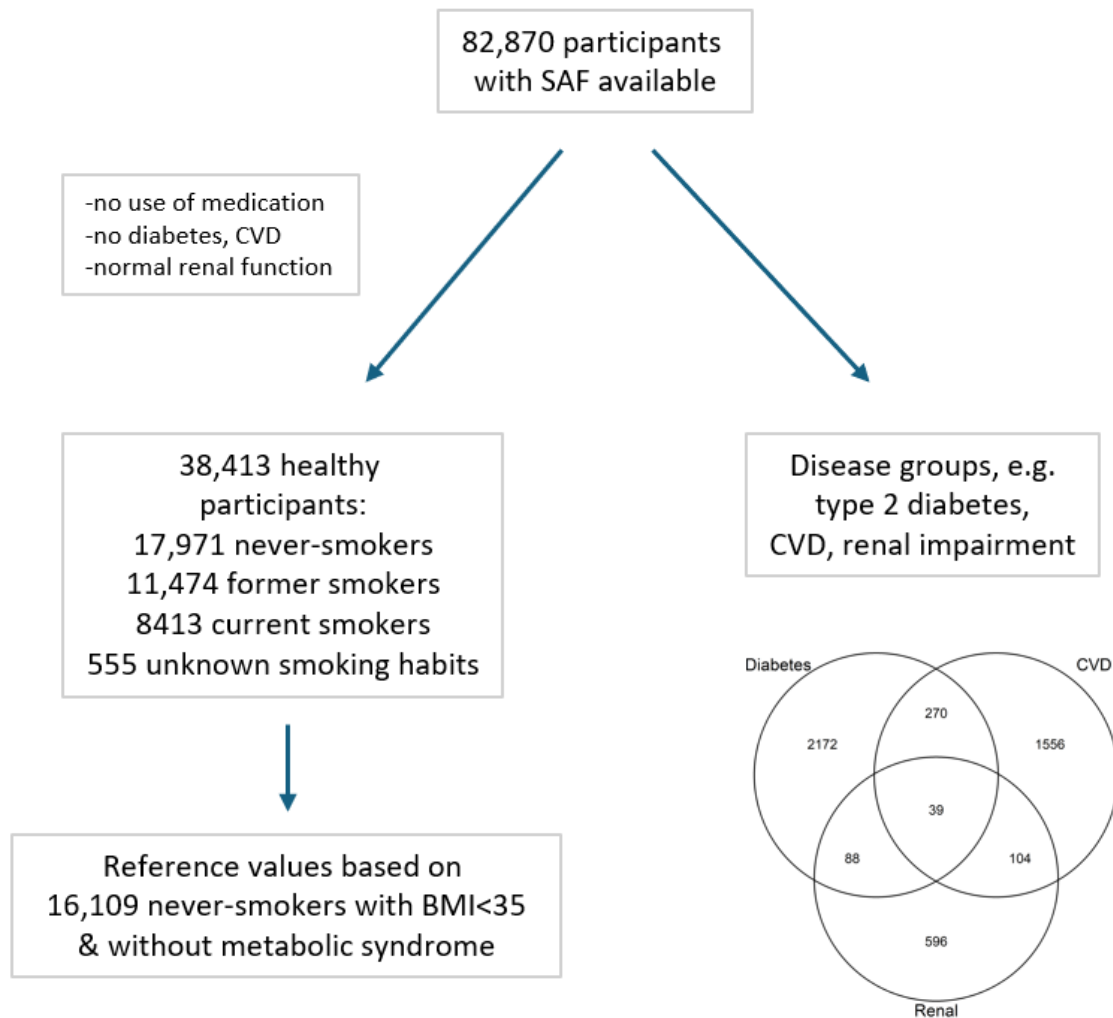

Supplement: Supplementary file 1 [file Image1.pdf]
