# Supplementary material for: Reference values and biological factors influencing skin autofluorescence
Source: Front Endocrinol (Lausanne). 2025 Nov 6;16:1700892. doi: 10.3389/fendo.2025.1700892 (PMC12631760; doi:10.3389/fendo.2025.1700892)
Supplement: Supplementary file 2 [file Image2.pdf]

Supplemental Figure 2.

The relationship between body mass index and expected age- and sex-adjusted SAF scores was evaluated using restricted cubic splines.

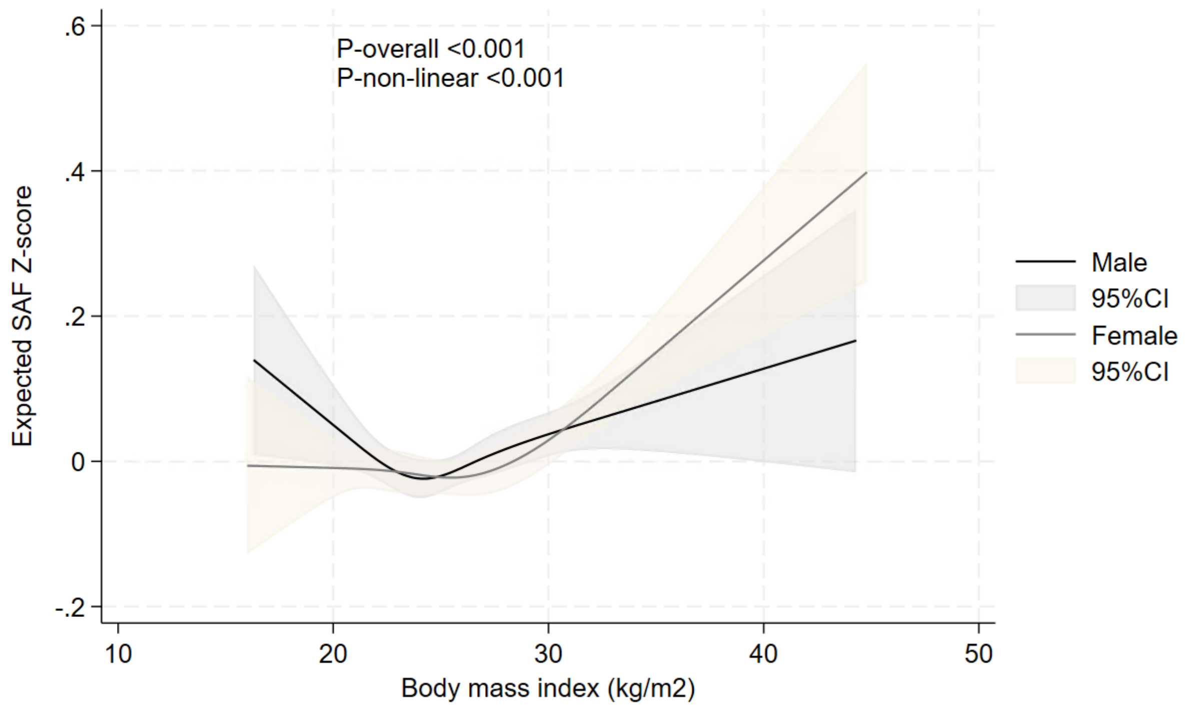

Legends:

The results are presented using solid black and grey lines for the expected SAF Z-score in males and females, respectively, and grey and light-brown shadow (indicating the 95% confidence interval) for the respective confidence intervals. P-values for non-linearity and overall association are also presented.
