# Supplementary material for: Reference values and biological factors influencing skin autofluorescence
Source: Front Endocrinol (Lausanne). 2025 Nov 6;16:1700892. doi: 10.3389/fendo.2025.1700892 (PMC12631760; doi:10.3389/fendo.2025.1700892)
Supplement: Supplementary file 3 [file Table1.docx]

**Supplemental Table 1.**

Baseline characteristics of Lifelines participants for whom SAF data were available or unavailable.

| Characteristic | With SAF | Without SAF |  |
| --- | --- | --- | --- |
| Sex (*n*; male/female) | 34551 / 48353  (41.7 / 58.3 %) | 28579 / 40697  (41.2 / 58.8 %) |  |
| Age (years) | 44.5 ± 12.6 | 44.9 ± 13.8 |  |
| BMI (kg/m^2^) | 26.1 ± 4.3 | 26.0 ± 4.4 |  |
| Waist circumference (cm) | 91 ± 12 | 90 ± 13 |  |
| Glucose (mmol/l) | 5.0 ± 0.8 | 5.0 ± 0.9 |  |
| HbA_1c_ (mmol/mol) | 37 ± 5 | 37 ± 5 |  |
| eGFR (ml/min/1.73m^2^) | 97 ± 15 | 96 ± 16 |  |
| Current smoking (%) | 21.5 | 21.3 |  |
| Former smoking (%) | 52.6 | 51.5 |  |
| Presence of type 2 diabetes (%) | 3.1 | 3.5 |  |
| Presence of CVD (%) | 2.4 | 3.0 |  |
| Presence of metabolic syndrome (%) | 13.9 | 12.4 |  |
| Presence of validated physical activity data (%) | 88.7 | 85.0 |  |
| Inclusion mode  Family doctor  Included family member  Self-registered | 56.6  30.7  12.7 | 49.6  34.5  15.9 |  |

Legends:

Data are presented as numbers, means ± SD, or percentages. BMI, body mass index; CVD, cardiovascular disease; eGFR, estimated glomerular filtration rate; HbA_1c_, glycated haemoglobin.
