# Supplementary material for: Reference values and biological factors influencing skin autofluorescence
Source: Front Endocrinol (Lausanne). 2025 Nov 6;16:1700892. doi: 10.3389/fendo.2025.1700892 (PMC12631760; doi:10.3389/fendo.2025.1700892)
Supplement: Supplementary file 4 [file Table2.docx]

**Supplemental Table 2.**

Baseline characteristics of Lifelines participants with available SAF, for whom detailed physical activity data were available or not available.

| Characteristic | With PA | Without PA |  |
| --- | --- | --- | --- |
| Sex (*n*; male/female) | 30,098 / 43,410  (40.9 / 59.1 %) | 4439 / 4923  (47.4 / 52.6 %) |  |
| Age (years) | 44.3 ± 12.2 | 46.0 ± 15.2 |  |
| BMI (kg/m^2^) | 26.1 ± 4.3 | 26.4 ± 4.5 |  |
| Waist circumference (cm) | 90 ± 12 | 92 ± 13 |  |
| Glucose (mmol/l) | 5.0 ± 0.8 | 5.1 ± 1.0 |  |
| HbA_1c_ (mmol/mol) | 37 ± 5 | 38 ± 6 |  |
| eGFR (ml/min/1.73m^2^) | 97 ± 15 | 95 ± 17 |  |
| Current smoking (%) | 21.3 | 21.4 |  |
| Former smoking (%) | 32.9 | 32.1 |  |
| Presence of type 2 diabetes (%) | 2.9 | 4.6 |  |
| Presence of CVD (%) | 2.2 | 4.1 |  |
| Presence of metabolic syndrome (%) | 13.7 | 14.9 |  |
| Inclusion mode  Family doctor  Included family member  Self-registered | 57.3  30.1  12.7 | 51.9  35.7  12.4 |  |

Legends:

Data are presented as numbers, means ± SD, or percentages. BMI, body mass index; CVD, cardiovascular disease; eGFR, estimated glomerular filtration rate; HbA_1c_, glycated haemoglobin.
